# *N*-Benzoyl-4-methyl­benzene­sulfonamide

**DOI:** 10.1107/S1600536810011967

**Published:** 2010-04-10

**Authors:** P. A. Suchetan, B. Thimme Gowda, Sabine Foro, Hartmut Fuess

**Affiliations:** a Department of Chemistry, Mangalore University, Mangalagangotri 574 199, Mangalore, India; b Institute of Materials Science, Darmstadt University of Technology, Petersenstrasse 23, D-64287 Darmstadt, Germany

## Abstract

In the title compound, C_14_H_13_NO_3_S, the N—H bond in is *anti* to the C=O bond. The dihedral angle between the two aromatic rings is 79.4 (1)°. In the crystal, mol­ecules are linked by N—H⋯O hydrogen bonds, generating *C*(4) chains.

## Related literature

For related structures, see: Gowda *et al.* (2009 ▶); Suchetan *et al.* (2010**a* ▶,b* ▶).
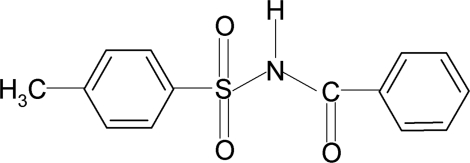


## Experimental

### 

#### Crystal data

C_14_H_13_NO_3_S
*M* *_r_* = 275.31
Orthorhombic, 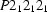

*a* = 5.1723 (5) Å
*b* = 14.785 (1) Å
*c* = 17.431 (1) Å
*V* = 1332.99 (17) Å^3^
*Z* = 4
Mo *K*α radiation
μ = 0.25 mm^−1^
*T* = 299 K
0.40 × 0.20 × 0.14 mm

#### Data collection

Oxford Diffraction Xcalibur diffractometer with a Sapphire CCD detector
Absorption correction: multi-scan (*CrysAlis RED*; Oxford Diffraction, 2009 ▶) *T*_min_ = 0.908, *T*_max_ = 0.966
5515 measured reflections
2644 independent reflections
2388 reflections with *I* > 2σ(*I*)
*R*_int_ = 0.013

#### Refinement

*R*[*F* ^2^ > 2σ(*F* ^2^)] = 0.033
*wR*(*F* ^2^) = 0.084
*S* = 1.12
2644 reflections
176 parameters
1 restraint
H atoms treated by a mixture of independent and constrained refinement
Δρ_max_ = 0.16 e Å^−3^
Δρ_min_ = −0.21 e Å^−3^
Absolute structure: Flack (1983 ▶), 1025 Friedel pairs
Flack parameter: 0.18 (8)

### 

Data collection: *CrysAlis CCD* (Oxford Diffraction, 2009 ▶); cell refinement: *CrysAlis RED* (Oxford Diffraction, 2009 ▶); data reduction: *CrysAlis RED* program(s) used to refine structure: *SHELXL97* (Sheldrick, 2008 ▶); molecular graphics: *PLATON* (Spek, 2009 ▶); software used to prepare material for publication: *SHELXL97*.

## Supplementary Material

Crystal structure: contains datablocks I, global. DOI: 10.1107/S1600536810011967/bt5236sup1.cif

Structure factors: contains datablocks I. DOI: 10.1107/S1600536810011967/bt5236Isup2.hkl

Additional supplementary materials:  crystallographic information; 3D view; checkCIF report

## Figures and Tables

**Table 1:** Hydrogen-bond geometry (Å, °)

*D*—H⋯*A*	*D*—H	H⋯*A*	*D*⋯*A*	*D*—H⋯*A*
N1—H1*N*⋯O2^i^	0.83 (2)	2.08 (2)	2.905 (2)	175 (2)

Symmetry code: (i) 
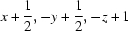
.

**Fig. 1.**
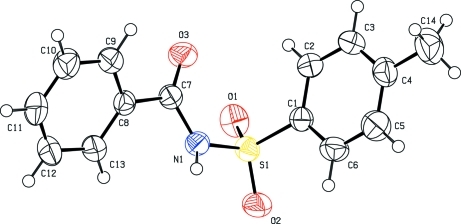
Molecular structure of the title compound, showing the atom-labelling scheme. Displacement ellipsoids are drawn at the 50% probability level.

**Fig. 2.**
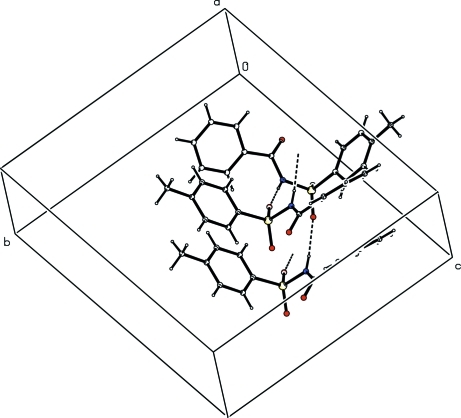
Molecular packing in the title compound. Hydrogen bonds are shown as dashed lines.

## supplementary crystallographic information

### Comment

Diaryl acylsulfonamides are known as potent antitumor agents against a broad spectrum of human tumor xenografts in nude mice. As a part of studying the effect of ring and the side chain substituents on the crystal structures of *N*-aromatic sulfonamides (Gowda *et al.*, 2009; Suchetan *et al.*, 2010*a,b*), the structure of *N*-(benzoyl)4-methylbenzenesulfonamide (I) has been determined. The conformation of the N—H bond in the C—SO_2_—NH—C(O) segment is *anti* to the C=O bond (Fig.1), similar to those observed in *N*-(benzoyl)benzenesulfonamide (II) (Gowda *et al.*, 2009), *N*-(benzoyl)4-chlorobenzenesulfonamide (III)(Suchetan *et al.*, 2010*b*) and *N*-(4-chlorobenzoyl)4-methylbenzenesulfonamide (IV)(Suchetan *et al.*, 2010*a*).

The dihedral angles between the sulfonyl benzene ring and the —SO_2_—NH—C—O segment is 76.5 (1)°, compared to the values of 86.5(0.1) in (II), 72.0 (1)° (molecule 1) and 77.3 (1)° (molecule 2) in (III), and 83.6 (1)° and 81.0 (1)° in the two independent molecules of (IV). Furthermore, the dihedral angle between the sulfonyl and the benzoyl benzene rings is 79.4 (1)°, compared to the vlues of 80.3(0.1) in (II), 62.8 (1)° (molecule 1) and 78.6 (1)° (molecule 2) in (III), and 81.0 (1)° and 76.3 (1)° in the two molecules of (IV).

The packing of molecules linked by of N—H···O(S) hydrogen bonds (Table 1) is shown in Fig. 2.

### Experimental

The title compound was prepared by refluxing a mixture of benzoic acid, 4-methylbenzenesulfonamide and phosphorous oxy chloride for 5 h on a water bath. The resultant mixture was cooled and poured into ice cold water. The solid, *N*-(benzoyl)4-methylbenzenesulfonamide obtained was filtered, washed thoroughly with water and then dissolved in sodium bicarbonate solution. The compound was later reprecipitated by acidifying the filtered solution with dilute HCl. The filtered and dried compound was recrystallized to the constant melting point.

Thick needle like colourless single crystals were grown from a slow evaporation of its toluene solution at room temperature.

### Refinement

The H atoms of the NH group was located in a difference map its coordinates were refined with a distance restraint of N—H = 0.86 (2)Å. The other H atoms were positioned with idealized geometry using a riding model with C—H = 0.93–0.96 Å. All H atoms were refined with isotropic displacement parameters set to 1.2 times of the *U*_eq_ of the parent atom.

### Crystal data

**Table:**

C_14_H_13_NO_3_S	*F*(000) = 576
*M**_r_* = 275.31	*D*_x_ = 1.372 Mg m^−^^3^
Orthorhombic, *P*2_1_2_1_2_1_	Mo *K*α radiation, λ = 0.71073 Å
Hall symbol: P 2ac 2ab	Cell parameters from 3284 reflections
*a* = 5.1723 (5) Å	θ = 2.7–27.9°
*b* = 14.785 (1) Å	µ = 0.25 mm^−^^1^
*c* = 17.431 (1) Å	*T* = 299 K
*V* = 1332.99 (17) Å^3^	Thick needle, colourless
*Z* = 4	0.40 × 0.20 × 0.14 mm

### Data collection

**Table:**

Oxford Diffraction Xcalibur diffractometer with a Sapphire CCD detector	2644 independent reflections
Radiation source: fine-focus sealed tube	2388 reflections with *I* > 2σ(*I*)
graphite	*R*_int_ = 0.013
Rotation method data acquisition using ω and phi scans.	θ_max_ = 26.4°, θ_min_ = 2.7°
Absorption correction: multi-scan (*CrysAlis RED*; Oxford Diffraction, 2009)	*h* = −3→6
*T*_min_ = 0.908, *T*_max_ = 0.966	*k* = −18→14
5515 measured reflections	*l* = −21→19

### Refinement

**Table:**

Refinement on *F*^2^	Secondary atom site location: difference Fourier map
Least-squares matrix: full	Hydrogen site location: inferred from neighbouring sites
*R*[*F*^2^ > 2σ(*F*^2^)] = 0.033	H atoms treated by a mixture of independent and constrained refinement
*wR*(*F*^2^) = 0.084	*w* = 1/[σ^2^(*F*_o_^2^) + (0.0398*P*)^2^ + 0.2729*P*] where *P* = (*F*_o_^2^ + 2*F*_c_^2^)/3
*S* = 1.12	(Δ/σ)_max_ = 0.009
2644 reflections	Δρ_max_ = 0.16 e Å^−^^3^
176 parameters	Δρ_min_ = −0.21 e Å^−^^3^
1 restraint	Absolute structure: Flack (1983), 1025 Friedel pairs
Primary atom site location: structure-invariant direct methods	Flack parameter: 0.18 (8)

### Special details

**Table:**

Experimental. CrysAlis RED (Oxford Diffraction, 2009) Empirical absorption correction using spherical harmonics, implemented in SCALE3 ABSPACK scaling algorithm.
Geometry. All esds (except the esd in the dihedral angle between two l.s. planes) are estimated using the full covariance matrix. The cell esds are taken into account individually in the estimation of esds in distances, angles and torsion angles; correlations between esds in cell parameters are only used when they are defined by crystal symmetry. An approximate (isotropic) treatment of cell esds is used for estimating esds involving l.s. planes.
Refinement. Refinement of *F*^2^ against ALL reflections. The weighted *R*-factor wR and goodness of fit *S* are based on *F*^2^, conventional *R*-factors *R* are based on *F*, with *F* set to zero for negative *F*^2^. The threshold expression of *F*^2^ > σ(*F*^2^) is used only for calculating *R*-factors(gt) etc. and is not relevant to the choice of reflections for refinement. *R*-factors based on *F*^2^ are statistically about twice as large as those based on *F*, and *R*- factors based on ALL data will be even larger.

### Fractional atomic coordinates and isotropic or equivalent isotropic displacement parameters (Å^2^)

**Table:**

	*x*	*y*	*z*	*U*_iso_*/*U*_eq_	
C1	0.4594 (4)	0.46253 (13)	0.51327 (11)	0.0386 (4)	
C2	0.5259 (5)	0.54024 (14)	0.55288 (13)	0.0515 (5)	
H2	0.4556	0.5520	0.6010	0.062*	
C3	0.6980 (5)	0.59989 (14)	0.52001 (14)	0.0594 (7)	
H3	0.7444	0.6518	0.5468	0.071*	
C4	0.8039 (4)	0.58456 (16)	0.44803 (14)	0.0538 (6)	
C5	0.7308 (6)	0.50747 (16)	0.40883 (15)	0.0622 (6)	
H5	0.7970	0.4965	0.3601	0.075*	
C6	0.5603 (5)	0.44644 (15)	0.44130 (14)	0.0530 (5)	
H6	0.5136	0.3945	0.4146	0.064*	
C7	0.5646 (4)	0.33550 (13)	0.67278 (11)	0.0385 (4)	
C8	0.7127 (4)	0.25923 (12)	0.70866 (10)	0.0372 (4)	
C9	0.9251 (5)	0.27975 (16)	0.75383 (12)	0.0493 (5)	
H9	0.9790	0.3394	0.7591	0.059*	
C10	1.0568 (5)	0.21114 (17)	0.79106 (14)	0.0611 (6)	
H10	1.2009	0.2250	0.8207	0.073*	
C11	0.9772 (5)	0.12268 (17)	0.78471 (13)	0.0573 (6)	
H11	1.0664	0.0770	0.8101	0.069*	
C12	0.7650 (5)	0.10229 (14)	0.74057 (13)	0.0557 (5)	
H12	0.7084	0.0428	0.7369	0.067*	
C13	0.6355 (5)	0.16987 (14)	0.70163 (12)	0.0479 (5)	
H13	0.4956	0.1553	0.6705	0.057*	
C14	0.9988 (6)	0.64902 (19)	0.41356 (17)	0.0819 (9)	
H14A	1.1659	0.6378	0.4355	0.098*	
H14B	0.9477	0.7102	0.4242	0.098*	
H14C	1.0065	0.6400	0.3591	0.098*	
N1	0.4428 (4)	0.31347 (11)	0.60414 (9)	0.0412 (4)	
H1N	0.496 (5)	0.2715 (12)	0.5770 (11)	0.049*	
O1	0.0820 (3)	0.42621 (11)	0.60730 (9)	0.0558 (4)	
O2	0.1517 (3)	0.32651 (11)	0.49499 (9)	0.0572 (4)	
O3	0.5488 (3)	0.40946 (10)	0.70188 (9)	0.0540 (4)	
S1	0.25282 (10)	0.38263 (3)	0.55513 (3)	0.04071 (13)	

### Atomic displacement parameters (Å^2^)

**Table:**

	*U*^11^	*U*^22^	*U*^33^	*U*^12^	*U*^13^	*U*^23^
C1	0.0395 (10)	0.0353 (9)	0.0411 (10)	0.0041 (9)	−0.0048 (9)	0.0016 (8)
C2	0.0721 (15)	0.0428 (11)	0.0396 (10)	−0.0038 (10)	−0.0006 (12)	−0.0037 (10)
C3	0.0835 (19)	0.0406 (12)	0.0540 (13)	−0.0141 (12)	−0.0158 (13)	0.0000 (10)
C4	0.0552 (14)	0.0489 (12)	0.0573 (13)	−0.0039 (10)	−0.0093 (11)	0.0136 (11)
C5	0.0709 (15)	0.0597 (13)	0.0559 (13)	−0.0026 (14)	0.0155 (14)	−0.0035 (11)
C6	0.0641 (13)	0.0448 (11)	0.0499 (12)	−0.0061 (11)	0.0065 (12)	−0.0104 (10)
C7	0.0408 (10)	0.0368 (10)	0.0380 (10)	0.0010 (9)	0.0040 (9)	−0.0001 (8)
C8	0.0409 (11)	0.0380 (9)	0.0328 (8)	0.0007 (8)	0.0044 (9)	0.0026 (7)
C9	0.0539 (13)	0.0453 (11)	0.0488 (12)	−0.0076 (11)	−0.0059 (11)	0.0037 (10)
C10	0.0593 (14)	0.0673 (16)	0.0568 (14)	−0.0063 (13)	−0.0160 (13)	0.0132 (12)
C11	0.0621 (14)	0.0585 (13)	0.0514 (12)	0.0065 (12)	−0.0053 (12)	0.0184 (11)
C12	0.0689 (14)	0.0400 (11)	0.0583 (13)	−0.0013 (13)	0.0011 (13)	0.0110 (9)
C13	0.0544 (12)	0.0422 (11)	0.0469 (11)	−0.0034 (10)	−0.0060 (10)	0.0047 (10)
C14	0.080 (2)	0.0802 (19)	0.0857 (19)	−0.0277 (16)	−0.0060 (17)	0.0271 (17)
N1	0.0501 (9)	0.0343 (8)	0.0392 (9)	0.0084 (8)	−0.0036 (8)	−0.0027 (7)
O1	0.0470 (8)	0.0542 (9)	0.0663 (10)	0.0119 (7)	0.0092 (8)	0.0055 (8)
O2	0.0612 (9)	0.0488 (8)	0.0615 (9)	−0.0100 (7)	−0.0204 (8)	−0.0014 (8)
O3	0.0706 (10)	0.0400 (8)	0.0515 (9)	0.0103 (8)	−0.0041 (8)	−0.0096 (7)
S1	0.0389 (2)	0.0368 (2)	0.0464 (3)	0.0022 (2)	−0.0042 (3)	0.0014 (2)

### Geometric parameters (Å, °)

**Table:**

C1—C6	1.379 (3)	C9—C10	1.384 (3)
C1—C2	1.384 (3)	C9—H9	0.9300
C1—S1	1.752 (2)	C10—C11	1.376 (3)
C2—C3	1.378 (3)	C10—H10	0.9300
C2—H2	0.9300	C11—C12	1.374 (4)
C3—C4	1.388 (3)	C11—H11	0.9300
C3—H3	0.9300	C12—C13	1.381 (3)
C4—C5	1.382 (3)	C12—H12	0.9300
C4—C14	1.512 (3)	C13—H13	0.9300
C5—C6	1.383 (3)	C14—H14A	0.9600
C5—H5	0.9300	C14—H14B	0.9600
C6—H6	0.9300	C14—H14C	0.9600
C7—O3	1.208 (2)	N1—S1	1.6556 (17)
C7—N1	1.391 (3)	N1—H1N	0.826 (15)
C7—C8	1.500 (3)	O1—S1	1.4224 (16)
C8—C9	1.385 (3)	O2—S1	1.4356 (16)
C8—C13	1.386 (3)		
			
C6—C1—C2	120.2 (2)	C11—C10—C9	120.8 (2)
C6—C1—S1	119.54 (16)	C11—C10—H10	119.6
C2—C1—S1	120.24 (16)	C9—C10—H10	119.6
C3—C2—C1	119.0 (2)	C12—C11—C10	119.5 (2)
C3—C2—H2	120.5	C12—C11—H11	120.2
C1—C2—H2	120.5	C10—C11—H11	120.2
C2—C3—C4	121.8 (2)	C11—C12—C13	120.3 (2)
C2—C3—H3	119.1	C11—C12—H12	119.9
C4—C3—H3	119.1	C13—C12—H12	119.9
C5—C4—C3	118.3 (2)	C12—C13—C8	120.4 (2)
C5—C4—C14	120.4 (2)	C12—C13—H13	119.8
C3—C4—C14	121.3 (2)	C8—C13—H13	119.8
C4—C5—C6	120.7 (2)	C4—C14—H14A	109.5
C4—C5—H5	119.7	C4—C14—H14B	109.5
C6—C5—H5	119.7	H14A—C14—H14B	109.5
C1—C6—C5	120.1 (2)	C4—C14—H14C	109.5
C1—C6—H6	120.0	H14A—C14—H14C	109.5
C5—C6—H6	120.0	H14B—C14—H14C	109.5
O3—C7—N1	122.85 (19)	C7—N1—S1	124.62 (14)
O3—C7—C8	122.68 (18)	C7—N1—H1N	121.2 (17)
N1—C7—C8	114.46 (16)	S1—N1—H1N	111.4 (16)
C9—C8—C13	119.17 (19)	O1—S1—O2	120.16 (11)
C9—C8—C7	118.51 (18)	O1—S1—N1	108.58 (9)
C13—C8—C7	122.21 (19)	O2—S1—N1	103.66 (9)
C10—C9—C8	119.8 (2)	O1—S1—C1	109.86 (9)
C10—C9—H9	120.1	O2—S1—C1	107.93 (10)
C8—C9—H9	120.1	N1—S1—C1	105.62 (10)
			
C6—C1—C2—C3	−1.4 (3)	C9—C10—C11—C12	0.3 (4)
S1—C1—C2—C3	177.07 (18)	C10—C11—C12—C13	1.3 (4)
C1—C2—C3—C4	0.7 (4)	C11—C12—C13—C8	−2.2 (4)
C2—C3—C4—C5	0.6 (4)	C9—C8—C13—C12	1.5 (3)
C2—C3—C4—C14	−178.1 (2)	C7—C8—C13—C12	−174.8 (2)
C3—C4—C5—C6	−1.2 (4)	O3—C7—N1—S1	−2.5 (3)
C14—C4—C5—C6	177.5 (3)	C8—C7—N1—S1	176.18 (15)
C2—C1—C6—C5	0.8 (4)	C7—N1—S1—O1	−44.6 (2)
S1—C1—C6—C5	−177.7 (2)	C7—N1—S1—O2	−173.41 (17)
C4—C5—C6—C1	0.6 (4)	C7—N1—S1—C1	73.21 (19)
O3—C7—C8—C9	−29.9 (3)	C6—C1—S1—O1	−152.71 (18)
N1—C7—C8—C9	151.34 (18)	C2—C1—S1—O1	28.8 (2)
O3—C7—C8—C13	146.3 (2)	C6—C1—S1—O2	−20.0 (2)
N1—C7—C8—C13	−32.4 (3)	C2—C1—S1—O2	161.54 (17)
C13—C8—C9—C10	0.1 (3)	C6—C1—S1—N1	90.38 (19)
C7—C8—C9—C10	176.5 (2)	C2—C1—S1—N1	−88.08 (18)
C8—C9—C10—C11	−1.0 (4)		

### Hydrogen-bond geometry (Å, °)

**Table:**

*D*—H···*A*	*D*—H	H···*A*	*D*···*A*	*D*—H···*A*
N1—H1N···O2^i^	0.83 (2)	2.08 (2)	2.905 (2)	175 (2)

Symmetry codes: (i) *x*+1/2, −*y*+1/2, −*z*+1.
